# Advances in oral treatment of inflammatory bowel disease using protein-based nanoparticle drug delivery systems

**DOI:** 10.1080/10717544.2025.2544689

**Published:** 2025-08-11

**Authors:** Zhihao Lin, Ziheng Zhao, Xianrui Lin, Zhenlin Yang, Lin Wang, Rui Xi, Dingpei Long

**Affiliations:** a State Key Laboratory of Resource Insects, Southwest University, Chongqing, China; b College of Sericulture, Textile and Biomass Sciences, Southwest University, Chongqing, China; c Westa College, Southwest University, Chongqing, China

**Keywords:** Inflammatory bowel disease, nanomedicine, protein-based nanoparticles, oral drug delivery, active targeting, stimuli-responsive delivery

## Abstract

Inflammatory bowel disease (IBD) comprises chronic autoimmune disorders with significant morbidity, highlighting the need for advanced, noninvasive, targeted therapies. Protein-based nanoparticle drug delivery systems (PNP-DDSs) have emerged as promising platforms to overcome limitations of conventional IBD therapies by improving drug stability and bioavailability while enabling colon-specific delivery. This review systematically classifies PNP-DDSs derived from natural proteins (albumin, gelatin, silk fibroin, and plant-derived proteins) and discusses their design principles along with strategies for intestinal targeting, including particle size and surface charge modulation, stimuli-responsive release (triggered by pH, reactive oxygen species, or enzymes), and active targeting. It highlights recent preclinical advances with oral PNP-DDSs delivering curcumin, resveratrol, 5-aminosalicylic acid, quercetin, and other anti-inflammatory agents, which demonstrate the therapeutic potential of these nanoplatforms in IBD models. Despite promising preclinical outcomes, clinical translation of PNP-DDSs remains challenging due to patient heterogeneity, manufacturing scale-up difficulties, and safety concerns. Future progress will require interdisciplinary innovation and optimization of multi‑stimuli-responsive designs for precise and safe clinical application of PNP-DDSs in IBD management.

## 1. Introduction

Inflammatory bowel disease (IBD) comprises a group of autoimmune disorders characterized by chronic intestinal inflammation. These conditions are marked by persistent inflammatory responses, disruption of the intestinal barrier, and an increased risk of developing colitis-associated neoplasms (Rubin et al. 2012; Francescone et al. 2015; Long 2024). Clinically, patients with IBD frequently experience gastrointestinal symptoms such as diarrhea, abdominal pain and weight loss, with some also presenting extraintestinal manifestations including arthritis, sclerosing cholangitis, and iritis (Rubin et al. 2012). Based on the anatomical site and pathological characteristics, IBD is primarily classified into Crohn’s disease (CD) and Ulcerative colitis (UC). CD can affect the entire gastrointestinal tract and is prone to complications like intestinal obstruction, whereas UC is generally confined to the colorectum, manifesting as hemorrhagic diarrhea and mucosal ulceration (Mowat et al. 2011; Rubin et al. 2012). Although IBD was once considered a predominantly Western disease, recent epidemiological data reveal a sharp increase in its incidence in newly industrialized regions such as South America, Asia and Africa, with the global prevalence now exceeding seven million cases. This globalization is attributed to various factors including environmental pollution, Westernized dietary patterns, antibiotic overuse, and dysbiosis of the intestinal microbiota (Ng et al. 2017; Ananthakrishnan et al. 2018).

Recent advancements in nano-drug delivery systems (NDDSs) have paved the way for novel therapeutic strategies for IBD (Patra et al. 2018). Nanoparticles (NPs), the core components of NDDSs, have been demonstrated to enhance drug bioavailability and prolong intestinal retention while reducing systemic toxicity owing to their high drug-loading capacity, controlled release properties, and improved targeting capabilities (Melo et al. 2018; Patra et al. 2018; Yang and Merlin 2019; Bhaskaran and Kumar 2021). Moreover, the capacity of NPs to modulate the gut microbiome and influence metabolic pathways further broadens their application in IBD management (Rajoka et al. 2021).

A variety of NDDSs, such as Poly(lactide-co-glycolide)/Eudragit S100 NPs (Makhlof et al. 2009), ε-polylysine-modified mesoporous silica (Nguyen et al. 2017), immunoliposomes (Harel et al. 2011), hydrogels (Xiao et al. 2014) and extracellular vesicles (Yang et al. 2010), are currently in preclinical development. Among these, protein-based nanoparticle drug delivery systems (PNP-DDSs) have garnered significant attention for oral IBD therapy due to the inherent advantages of natural proteins, including biodegradability, low immunogenicity, and efficient target modification capabilities (Lohcharoenkal et al. 2014; Jain et al. 2018; Zhang and Merlin 2018; Shah et al. 2020). In contrast to conventional routes, such as rectal or intravenous administration, oral PNP-DDSs can effectively circumvent drug inactivation by gastric acid and digestive enzymes. Furthermore, these systems can be engineered to target the colon specifically by modulating particle size, pH responsiveness, and surface charge. Such refinements not only improve patient compliance but also enhance therapeutic precision, thereby leading to more favorable treatment outcomes.

Compared to conventional polymeric or liposomal NDDSs, PNP-DDSs exhibit several distinctive strengths beyond their inherent biocompatibility. For example, they can achieve functional synergy: lactoferrin (LF), as a carrier, not only facilitates drug loading but also releases degradation products with additional therapeutic benefits (Li et al. 2024). The allosteric nature of protein structures enables the design of intelligent, stimuli-responsive release systems that promote precise drug delivery, even though proteins can be somewhat sensitive to degradation in the gastrointestinal environment (Liu et al. 2024). Furthermore, the active sites on amino acid residues allow for efficient drug binding, and surface groups are readily modifiable with targeting ligands, thereby enhancing delivery specificity and efficiency. While PNP-DDSs may face certain challenges, such as generally moderate drug-loading capacity, some degree of batch-to-batch variability due to the natural origin of proteins, and potentially higher production costs at scale, many of these limitations can be mitigated through advances in protein engineering, process optimization, and formulation technology. Overall, the unique multifunctional properties of PNP-DDSs continue to drive innovation and expand their potential in advanced drug delivery applications.

Currently, most reviews on PNP-DDSs emphasize their broad applications, with limited focus on oral therapies specifically for IBD. Likewise, existing reviews on oral IBD treatment, while discussing other nanosystems such as liposomes, rarely provide a systematic summary of PNPs as promising carriers. This gap indicates that the unique potential of PNPs remains underexplored and thus serves as the rationale for this review. Here, we systematically examine the classification and design principles of PNP-DDSs, including albumin-, gelatin-, and silk protein-based nanoparticles, and their intestinal targeting strategies. We further highlight recent advances in the oral administration of PNP-DDSs loaded with therapeutic agents such as curcumin (CUR) and 5-aminosalicylic acid (5-ASA) in animal models of IBD. Additionally, we discuss future challenges, including the need for personalized therapeutic approaches, the integration of multi-stimuli responsive systems, and the development of plant-derived protein carriers. The goal of this review is to provide a solid theoretical foundation for future innovations in the clinical treatment of IBD.

## 2. Classification of PNP-DDSs

**Figure 1.**
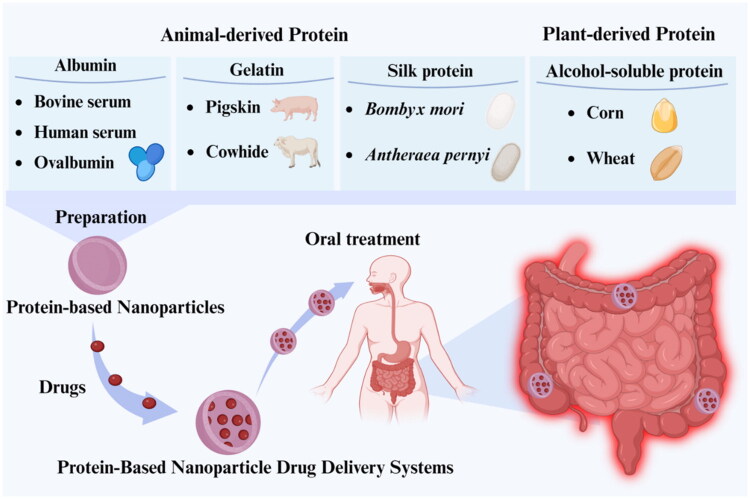
Protein classification and mode of administration of PNP-DDSs. Proteins extracted from animals and plants are used as raw materials to prepare NPs. Based on NPs derived from different protein sources, PNP-DDSs are further constructed to achieve oral targeted therapy. Created in https://BioRender.com.

PNP-DDSs are categorized into four primary groups based on differences in protein origin and functional properties: albumin-based, gelatin-based, silk protein-based, and alcohol-soluble protein-based NPs (Figure 1). The following sections discuss the design strategies for these various PNP-DDSs and their potential applications in the oral treatment of IBD.

### 2.1. Albumin-based NPs

Albumin-based NPs (ANPs) are typically synthesized from natural albumins such as bovine serum albumin (BSA), human serum albumin (HSA), and ovalbumin (Lohcharoenkal et al. 2014). Due to structural and biochemical differences among these albumins, the resulting NPs exhibit distinct properties and application potentials in drug delivery.

BSA NPs are composed of three homologous domains, each further divided into two subdomains. The principal drug-binding sites reside in domains IIA and IIIA, which can effectively accommodate fatty acids, therapeutic compounds, and metal ions (Kubiak-Ossowska et al. 2019; Szymaszek et al. 2022). Owing to their excellent biocompatibility, biodegradability, and low immunogenicity, BSA NPs are widely used for drug delivery, particularly in intestinal administration, where they enhance drug retention and support mucosal barrier integrity (Elzoghby et al. 2012a; Spada et al. 2021; Duan et al. 2022; Yu et al. 2022).

HSA NPs consist of three highly conserved domains, each composed of six α-helices. Their primary ligand-binding sites are also located in domains IIA and IIIA, enabling binding to both endogenous molecules (e.g. bilirubin, fatty acids) and exogenous therapeutic agents (Elzoghby et al. 2012a; Tao et al. 2021). Salient features of HSA, including extremely low immunogenicity, high biodegradability, minimal toxicity, and wide availability, make it a promising vehicle for intestinal drug delivery (Fasano et al. 2005; Kratz 2008).

Ovalbumin NPs are characterized by four free sulfhydryl groups and one disulfide bond and consist mainly of α-helices and β-sheets. Their hydrophobic core serves as a drug-binding region suitable for encapsulating lipophilic compounds and small-molecule drugs (Strixner and Kulozik 2011; Vesković et al. 2022). Moreover, ovalbumin exhibits sensitivity to pH and temperature, a trait that renders it a potential candidate for intestinal targeting and inflammation-responsive drug release, though its applications remain underexplored (Wongsasulak et al. 2010).

The advantages of ANPs can be summarized in three key aspects. First, they offer excellent drug-loading capabilities: the NP surfaces are rich in charged amino acids and functional groups that enable efficient encapsulation of both hydrophilic and hydrophobic drugs via electrostatic adsorption or covalent conjugation (Kratz 2008). Second, the abundance of reactive groups, primarily amino and carboxyl moieties, facilitates targeted modification by coupling with specific ligands such as folic acid (FA) and antibodies, thereby enabling site-specific drug delivery (Tang et al. 2011). Third, ANPs are naturally biodegradable, exhibit low immunogenicity, and can be readily fabricated using scalable techniques such as desolvation, controlled coacervation, or emulsification, making them suitable for industrial production (Elzoghby et al. 2012a).

### 2.2. Gelatin-based NPs

Gelatin, a natural polymer derived from collagen, is typically extracted from animal tissues such as pig skin and cowhide through acid, alkaline, or enzymatic hydrolysis. Gelatin obtained via acid treatment is classified as type A, while that derived through alkaline processing is referred to as type B (Elzoghby et al. 2012b; Foox and Zilberman 2015; Jain et al. 2018). Due to increasing global concerns related to religious dietary restrictions, the rise of vegetarianism, and prion-associated risks, alternative gelatin sources have emerged, including poultry, fish, and other vertebrates (Foox and Zilberman 2015).

Gelatins from different sources exhibit distinct physicochemical properties. For example, fish-derived gelatin differs significantly from mammalian gelatin in its amino acid composition and displays a lower melting point, which compromises thermal stability and functional performance at human body temperature. Additionally, it may induce allergic responses in sensitive individuals (Aas 1966). To address the limitations associated with animal-derived gelatin, the development of recombinant gelatin has gained increasing attention (Olsen et al. 2003).

Structurally, gelatin contains a high density of functional groups, such as amino, carboxyl, and hydroxyl moieties, and features a loose three-dimensional network. These characteristics enable efficient drug loading via multiple mechanisms, offering broad compatibility for various therapeutic agents. Moreover, the chemical similarity between gelatin and native human collagen results in low immunogenicity, while its capacity for facile chemical cross-linking further enhances drug entrapment, particularly for hydrophobic molecules (Elzoghby 2013; Lohcharoenkal et al. 2014).

As a well-established biomaterial with a history of safe use in pharmaceutical and medical applications, gelatin has been widely investigated for NDDSs. Gelatin-based NPs (GNPs) offer several advantageous features, including controlled drug release, surface modifiability, and site-specific accumulation at inflamed tissues. Their drug release kinetics can be tailored by modifying preparation parameters, such as the isoelectric point, to adapt to various inflammatory microenvironments (Jain et al. 2018). Furthermore, release profiles can be fine-tuned by altering the gelatin source, molecular weight, and cross-linking degree (Young et al. 2005).

GNPs also facilitate the delivery of nucleic acid therapeutics and hydrophobic anti-inflammatory agents through ionic interactions or polyethylene glycol modification, which additionally extends systemic circulation time (Zillies and Coester 2005; Narayanan et al. 2013). Importantly, GNPs exhibit preferential accumulation in macrophage-rich regions, such as intestinal inflammatory sites, thereby increasing local drug concentrations and enhancing therapeutic efficacy (Jain et al. 2018).

### 2.3. Silk protein-based NPs

Currently, silk proteins used as nanocarriers are mainly derived from *Bombyx mori* (domesticated silkworm) and *Antheraea pernyi* (wild silkworm) (Silva et al. 2019). Silk protein from *B. mori* primarily consists of silk fibroin (SF, ∼75%) surrounded by silk sericin (SS, ∼25%). Historically, SS was considered industrial waste and was routinely discarded in large quantities. Recent studies, however, have revealed that SS possesses excellent biocompatibility, along with antioxidant properties, pH sensitivity, and inhibitory activities against elastase and tyrosinase, leading to its widespread application in biomedical fields (Das et al. 2021). Nevertheless, research exploring *B. mori* SS-based NPs (BmSSNPs) as oral drug delivery systems for IBD remains limited. To date, only a few studies have demonstrated that BmSSNPs can stably encapsulate water-insoluble drugs (Wang, Li, et al. 2022), and complexes formed by combining BmSSNPs with probiotics can effectively prolong drug retention in inflamed intestinal tissues (Zhang et al. 2025).

Degummed natural *B. mori* cocoon silk yields SF fibers characterized by a stable antiparallel *β*-sheet crystalline structure, which endows the silk with exceptional mechanical strength and toughness (Jain et al. 2018; Hong et al. 2020). When these fibers are regenerated and dissolved to produce *B. mori* SF-based NPs (BmSFNPs), the resulting particles exhibit robust self‑assembly and maintain their structural integrity across a broad pH range, even under high salt concentrations or in the presence of metal ions, thereby conferring inherent advantages for gastrointestinal drug delivery (Yadav et al. 2024). These NPs are highly biocompatible, showing minimal inflammatory or coagulation responses, which supports their potential for both systemic and localized therapeutic applications (Jain et al. 2018; Yadav et al. 2024). Furthermore, BmSFNPs can autonomously respond to external stimuli, such as changes in pH or elevated reactive oxygen species (ROS) levels, to trigger drug release without necessitating additional chemical modifications. Their high drug-loading capacity and controlled release profiles contribute to minimizing off-target effects (Seib et al. 2013; Mottaghitalab et al. 2015). Targeting strategies have further enhanced colonic accumulation by incorporating magnetic NPs (Tian et al. 2014) or by modifying the NP surface to target CD44 receptors (Gou et al. 2019).

Compared to BmSFNPs, *A. pernyi* SF-based NPs (ApSFNPs) exhibit multiple responsive characteristics to pH, ROS, and glutathione. Furthermore, ApSFNPs are enriched with Arg-Gly-Asp (RGD) tripeptide sequences, enabling them to specifically recognize and bind integrin receptors highly expressed on the membranes of epithelial cells and macrophages in inflamed colonic tissues, thus actively targeting inflammatory sites (Lu et al. 2014). Additionally, ApSFNPs possess efficient lysosomal escape capabilities, significantly enhancing their drug delivery efficiency and therapeutic efficacy (Ma et al. 2022).

### 2.4. Alcohol soluble protein-based NPs

Alcohol soluble proteins, derived from botanical sources such as wheat or corn, are characterized by their pronounced hydrophobic properties. Zein, a strongly hydrophobic protein known for its natural barrier penetration capabilities, is commonly utilized to fabricate zein-based NPs (ZNPs). These NPs can encapsulate hydrophobic drugs within their core and resist degradation by gastric acid and digestive enzymes, thereby significantly improving drug bioavailability (Zou et al. 2012; Hashem et al. 2015). Moreover, ZNPs exhibit slow and controlled drug release characteristics, a critical attribute in pharmaceutical research. As they gradually degrade in the intestinal tract, ZNPs extend the duration of drug release, which reduces the frequency of dosing and enhances patient compliance (Zou et al. 2012; de Almeida Campos et al. 2023). In addition, ZNPs have demonstrated potential in metabolic regulation. Studies suggest that they can synergistically alleviate IBD symptoms by modulating short-chain fatty acid metabolism and restoring intestinal flora balance (Yang, Chen, et al. 2024).

### 2.5. Comparison of representative PNP-DDSs

PNP-DDSs exhibit distinct advantages in drug delivery owing to their varied biological sources and structural characteristics (Table 1). ANPs offer low immunogenicity and versatile drug-loading capacities across a broad spectrum of therapeutic agents. GNPs are adaptable to diverse applications due to their flexible sourcing and tunable release profiles. Silk protein-based NPs, particularly those derived from ApSFNPs, are notable for their excellent structural stability and stimuli-responsive behavior, demonstrating significant promise in active targeting applications. Alcohol soluble protein-based NPs display potential in the delivery of hydrophobic drugs and in metabolic regulation. However, each system has its limitations, which may include constraints related to source availability, insufficient targeting specificity, or limited research maturity. Future developments may focus on structural modifications, surface engineering, and optimization of fabrication techniques to further enhance their therapeutic efficacy and expand their clinical applicability.

**Table 1.** Comparison of representative PNP-DDSs.

Protein-based NPs	Category	Structural features	Advantages	Limitations	Representative applications
Albumin-based NPs	BSA NPs	Three homologous domains with subdomains Binding sites located in domains IIA and IIIA	Excellent biocompatibility and biodegradability Low immunogenicity Enhance intestinal drug retention High encapsulation efficiency Surface modifiability for targeting	Slightly higher immunogenicity than HSA Limited pH and temperature responsiveness	Broad drug delivery, particularly for intestinal administration
	HSA NPs	Three conserved domains composed of α-helices Ligand-binding sites in domains IIA and IIIA	Extremely low immunogenicity High biodegradability and biocompatibility Low toxicity and wide availability Strong drug-loading capacity Modifiable for lesion-specific targeting	Source limited to human serum Weak responsiveness to stimuli	Intestinal drug delivery Site-specific delivery via folate or antibody modification
	OVA NPs	Contain free sulfhydryl groups and disulfide bonds Composed of α-helices and β-sheets Hydrophobic core for drug binding	Suitable for lipophilic and small-molecule drugs Responsive to pH and temperature Low immunogenicity and degradability	Limited research maturity Weak universality in drug loading Targeting needs optimization	Intestinal targeted delivery Inflammation-responsive therapy
Gelatin-based NPs	Type a/B and Alternative source	Contains amino, carboxyl, hydroxyl groups Loose 3D network Fish gelatin has low melting temperature	Broad drug-loading compatibility Low immunogenicity Easy crosslinking Tunable release kinetics Suitable for nucleic acid and anti-inflammatory delivery Prolonged half-life Preferential accumulation in macrophage-rich areas	Source limitations due to religious and dietary restrictions Fish gelatin has poor thermal stability and potential allergenicity	Delivery of nucleic acids and anti-inflammatory agents Intestinal inflammation-targeted therapy
Silk protein-based NPs	BmSFNPs	Antiparallel β-sheet structure Heavy/light chains linked by disulfide bonds Stable under pH and salt stress	High structural stability Minimal immune and clotting response High drug-loading and controlled release Surface modifiable for targeting	Limited research on oral IBD delivery Targeting relies on external ligands	Gastrointestinal and systemic/local drug delivery
	ApSFNPs	Rich in arginine and RGD sequences Contains α-helices and β-sheets Capable of lysosomal escape	Strong integrin-mediated active targeting Multi-stimulus responsiveness Improved intracellular delivery Excellent biocompatibility	Limited research scale Production standardization needed Potential off-target effects	Active targeting for intestinal inflammation Enhanced intracellular drug delivery
Alcohol soluble protein-based NPs	ZNPs	Plant-derived, rich in hydrophobic amino acids Predominantly irregular coils and β-sheets Hydrophobic core with limited functional groups	Excellent mucosal penetration Stable in gastric acid, enhancing bioavailability Controlled and sustained release Potential to regulate metabolism and gut microbiota	Poor encapsulation of hydrophilic drugs Limited drug-loading flexibility Source limitation	Oral delivery of hydrophobic drugs Adjuvant therapy for IBD via microbiota modulation

## 3. Colonic targeting design strategies for oral PNP-DDSs

**Figure 2.**
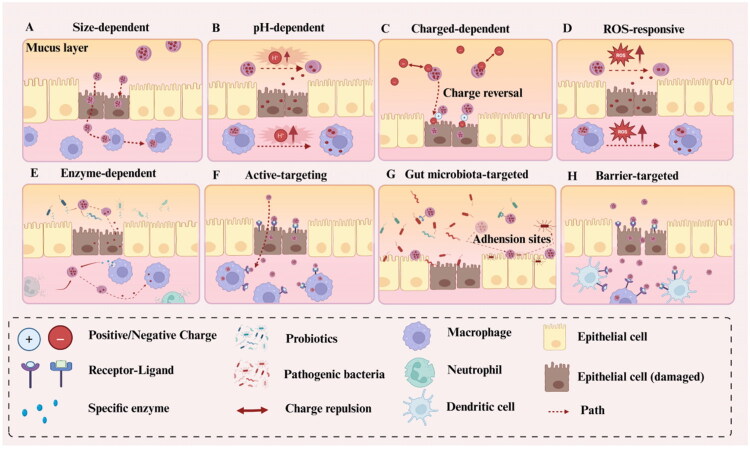
Schematic illustration of various NP-based targeting strategies. (**A**) Size-dependent targeting: Larger NPs are more likely to be trapped within the mucus layer, while smaller NPs exhibit enhanced penetration and are more efficiently internalized by epithelial cells and macrophages. (**B**) pH-dependent targeting: NPs are designed to release their therapeutic payload in response to pH variations either in the extracellular environment or within macrophages following endocytosis. (**C**) Charged-dependent targeting: NPs initially possess a negative charge to facilitate penetration through the negatively charged mucus layer. Upon approaching epithelial cells, a charge reversal (to positive) enhances their interaction with negatively charged cell membranes. (**D**) ROS-responsive targeting: Elevated levels of ROS at inflamed sites trigger the degradation of ROS-sensitive moieties in NPs, resulting in controlled drug release. (**E**) Enzyme-responsive targeting: NPs are hydrolyzed by disease-specific enzymes present in the Colon, enabling localized drug release. (**F**) Active targeting: NPs are functionalized with ligands that selectively bind to overexpressed receptors on epithelial cells or immune cells, such as macrophages. (**G**) Gut microbiota-targeted delivery: NPs recognize microbial surface antigens or receptors, or compete with pathogens for epithelial adhesion sites, modulating host–microbiota interactions. (**H**) Barrier-targeted strategy: NPs facilitate intestinal barrier repair by targeting specific receptors expressed on epithelial cells, macrophages, or dendritic cells. Created in https://BioRender.com.

Oral PNP-DDSs have emerged as the preferred therapeutic modality for IBD because of their noninvasive administration, high patient compliance, and ability to specifically target the colon, advantages that surpass those offered by rectal or intravenous routes. The integration of nanotechnology into these systems not only protects drugs from degradation in the gastrointestinal tract but also extends their retention at the target site. Moreover, these delivery platforms can be engineered to release therapeutic agents in response to the inflammatory microenvironment, thereby enhancing treatment efficacy while reducing systemic toxicity. As illustrated in Figure 2, the following sections elaborate on eight key design strategies that underlie the development of effective colonic-targeted oral PNP-DDSs.

### 3.1. Size-dependent targeting

NPs size is a critical determinant of intestinal penetration, targeting specificity and uptake by immune cells. Research indicates that PNP-DDSs with particle sizes between 100 and 200 nm exhibit enhanced tissue penetration, efficient immune targeting, and reduced clearance by scavenger mechanisms. Given that the pore size of the intestinal mucus layer ranges from 50 to 200 nm, maintaining particle sizes <200 nm, especially when combined with polyethylene glycol modification, can significantly decrease mucus retention and improve penetration at the lesion site (Lamprecht et al. 2005; Li et al. 2022; Yasmin et al. 2022). Additionally, immune cells such as macrophages more readily internalize particles <500 nm, thereby facilitating the targeted delivery of anti-inflammatory agents and enhancing therapeutic outcomes (Xiao and Merlin 2012; Yasmin et al. 2022). Smaller NPs are also better equipped to withstand the rapid intestinal transit caused by diarrhea, which prolongs their therapeutic window. Thus, precise control over NPs size is essential for optimizing drug delivery efficiency.

### 3.2. pH-dependent targeting

The gastrointestinal tract of patients with IBD exhibits a distinct pH gradient, approximately 2 in the gastric region, from 6.8 to 8 in the ileum, and from 5.3 to 7.5 in the colon (Zeeshan et al. 2019). pH-dependent PNP-DDSs exploit this gradient to achieve colon-specific drug release. One strategy involves the use of enteric coatings, such as Eudragit-S100, which shield the drug from gastric acid and pH fluctuations, ensuring that the drug disintegrates and is released specifically in the colonic environment. This enhances both the stability and bioavailability of the therapeutic agent (Makhlof et al. 2009). Another strategy leverages BmSFNPs for their ability to mediate precise controlled release: these systems exhibit a low release rate under neutral conditions (pH 7.4) that increases significantly under acidic conditions, thereby extending drug retention in the intestine (Seib et al. 2013). Finally, the development of multi-threshold response systems is crucial to accommodate individual patient differences, disease states, and feeding conditions. For example, since colonic pH in patients with active UC can drop to as low as 5.5, a dual sensitivity system operating between pH 5.5 and 7.0 can be designed to ensure precise and responsive drug release (Zeeshan et al. 2019).

### 3.3. Charged-dependent targeting

The colonic mucosal surface in IBD patients is rich in negatively charged mucins (e.g. MUC2), which naturally serve as anchors via charge interactions. Utilizing negatively charged PNP-DDSs with zeta potentials ranging from −20 to −30 mV exploits electrostatic repulsion to minimize nonspecific adhesion to the mucosa. This strategy enhances permeability in inflamed tissues and prolongs drug retention and absorption at the target site (Zhang et al. 2021; Li et al. 2022). Furthermore, NPs can be engineered to undergo charge switching through phosphate modification. This modification allows them to maintain a negative charge within the mucus layer and subsequently transition to a positive charge once they traverse the mucus, thereby synergistically promoting enhanced penetration and uptake by epithelial cells (Han et al. 2012; Veider et al. 2024). In contrast, NPs that are positively charged from the outset may precipitate premature drug release, reducing therapeutic efficacy. Combining charge modification with active targeting ligands, such as anti-mucosal vascular addressin cell-adhesion molecule-1 (anti-MAdCAM-1) antibodies, can further increase drug accumulation in the colon by up to 2.8-fold (Li et al. 2023). Consequently, negative charge modification has emerged as a preferred approach for optimizing targeted drug delivery (Jacob et al. 2020; Li et al. 2022).

### 3.4. ROS-responsive targeting

Inflammatory sites in IBD typically exhibit ROS levels that are 10–100 times higher than those in normal tissues, presenting a unique opportunity for the development of ROS-responsive PNP-DDSs (Jacob et al. 2020). By incorporating ROS-sensitive moieties (e.g. sulfide, ether bonds, or phenylboronic esters) into the NP matrix, these systems can trigger rapid drug release via chemical bond cleavage under high ROS conditions (Chen et al. 2017; Jacob et al. 2020). Moreover, PNP-DDSs loaded with antioxidants like resveratrol (RES) can simultaneously scavenge excess ROS and deliver anti-inflammatory agents, thereby achieving a dual synergistic effect of antioxidant and anti-inflammatory actions (Lozano-Pérez et al. 2014; Gou et al. 2019). Additionally, integrating magnetic BmSFNPs with an ROS-responsive coating enables magnetic targeting aggregation at the site of inflammation. This configuration allows drug release to be finely tuned according to local ROS levels, thereby providing a spatiotemporally coordinated therapeutic effect (Tian et al. 2014; Gou et al. 2019).

### 3.5. Enzyme-dependent targeting

In the inflammatory milieu characteristic of IBD, the concentrations of enzymes, such as *β*-glucosidase, cellulase, azoreductase, nitroreductase and myeloperoxidase (MPO), are markedly elevated in the colon. These enzymes may originate from colonic microorganisms or be released by activated neutrophils at inflammatory sites (Liu, Dong, et al. 2021; Li et al. 2022). By employing substrates that are selectively cleaved by these enzymes as carrier materials, PNP-DDSs can be engineered to trigger drug release specifically at the site of inflammation. Furthermore, to safeguard the stability of PNP-DDSs in the harsh acidic conditions of the stomach, polymeric materials such as chitosan, dextran, and pectin can be incorporated. These materials remain stable in gastric acid yet are degraded by specific colonic enzymes, ensuring that drug release occurs precisely at the inflammatory locus, thereby facilitating targeted therapy (Li et al. 2022). Additionally, biomaterials like HSA or hemoglobin, which interact with MPO, can be utilized to further refine targeted delivery by enabling the nanosystems to respond selectively to high MPO levels at the site of inflammation.

### 3.6. Active-targeting strategies

In IBD patients, colonic epithelial cells and activated macrophages frequently overexpress a variety of specific receptors, which underpins the rationale for designing PNP-DDSs based on active-targeting strategies (Liu, Dong, et al. 2021). Commonly targeted receptors include CD44 and the FA receptor (Gou et al. 2019; Ye et al. 2023). For instance, systems targeting the FA receptor facilitate drug accumulation in inflamed regions by specifically binding to activated macrophages (Ye et al. 2023). Similarly, chondroitin sulfate modifications that target the CD44 receptor, overexpressed on both macrophages and epithelial cells, significantly enhance cellular uptake through receptor-mediated endocytosis (Gou et al. 2019). This active-targeting approach has been demonstrated to not only improve therapeutic efficacy but also reduce systemic toxicity by ensuring that higher concentrations of the drug are delivered directly to the inflamed tissue.

### 3.7. Gut microbiota-targeted strategies

IBD is considered a consequence of disrupted interactions between the host and intestinal microbiota. Patients with IBD typically exhibit significant gut microbial dysbiosis, characterized by reduced microbial diversity, decreased abundance of beneficial bacteria, and increased populations of pathogenic species (Qiu et al. 2022). This dysbiosis exacerbates IBD progression through mechanisms such as metabolic disturbances, impaired intestinal barrier function, and aberrant immune activation. There is a bidirectional interaction between PNPs and the intestinal microbiota. On the one hand, PNPs can influence microbiota composition positively by producing polypeptides or amino acids upon degradation that promote the growth of beneficial bacteria, or by competitively inhibiting pathogenic bacteria adhesion to intestinal epithelial surfaces. On the other hand, gut microbial metabolites may affect the stability and integrity of PNPs or indirectly alter their transport and delivery efficiency by modifying the intestinal microenvironment. Precise targeting of microbiota can be enhanced by surface modification of PNPs with ligands that specifically recognize microbial surface antigens (e.g. lipopolysaccharides, capsular polysaccharides) or receptors (e.g. glycoproteins, fimbrial proteins), or by conjugating PNPs with phage-derived tail fibers capable of selective microbiota recognition. For instance, LF NPs release bioactive peptides upon degradation that exhibit anti-inflammatory, antimicrobial, immunomodulatory, and epithelial protective effects, all contributing to their therapeutic efficacy in IBD (Li et al. 2024). Additionally, the use of germ-free mouse models in research can help distinguish between the direct biological effects of PNPs and those mediated indirectly through the microbiota.

### 3.8. Barrier-targeted strategies

Patients with IBD exhibit significant structural and functional disruptions in the intestinal barrier, comprising the chemical barrier formed by the mucus layer, the physical barrier constituted by the epithelial cell layer, and the immune barrier involving various immune cells and cytokines (Camilleri 2019). Structurally, inflammatory mediators disrupt tight junctions between epithelial cells, promoting cell apoptosis and mucosal ulceration, thereby exacerbating barrier damage. Functionally, these changes result in increased epithelial permeability, mucus layer thinning, and impaired immune cell function. Critically, gut microbiota metabolites permeating through the compromised barrier can further activate immune cells, driving additional inflammatory responses and establishing a vicious cycle of "barrier damage–inflammation amplification–further damage." The structural and functional integrity of the intestinal barrier relies on complex interactions among diet, gut microbiota, and intestinal immune or stromal cells (Iacucci et al. 2024). This interplay offers promising therapeutic targets for individualized IBD treatment, with PNP-DDSs presenting significant advantages. For instance, HSA NPs facilitate endocytosis via gp60 receptors expressed on intestinal epithelial cells, aiding barrier repair. GNPs can promote epithelial cell proliferation and restore tight junction integrity, directly contributing to barrier restoration. Furthermore, therapeutic outcomes can be further improved by surface-modifying NPs with targeting ligands: RGD peptides can specifically target integrins that are overexpressed on epithelial and vascular endothelial cells in IBD; mannose moieties can target mannose receptors abundant on intestinal macrophages and dendritic cells. Notably, strategies aimed at repairing intestinal barrier dysfunction and breaking the inflammatory vicious cycle have emerged as critical components in the effective management of IBD.

In summary, the complexity of the gastrointestinal environment necessitates the strategic integration of multiple targeting approaches. By precisely controlling NP fabrication parameters, conjugating specific targeting ligands, and optimizing surface modifications, multi-mechanistic and synergistic precision delivery systems can be developed to significantly enhance therapeutic outcomes. Table 2 provides a comprehensive summary and comparison of the advantages and limitations of various targeting strategies.

**Table 2.** Comparative summary of targeting strategies for oral PNP-DDSs in IBD therapy.

Targeting strategy	Core principle	Advantages	Limitations
Size-dependent	Particle size (100–200 nm optimal) regulates intestinal penetration, immune uptake, and retention.	Facilitates mucus penetration Improves intestinal retention Reduces rapid clearance	Requires strict size control (±10 nm), complicating large-scale production Particles <50 nm are prone to renal clearance; >200 nm are captured by the RES
pH-dependent	Leverages gastrointestinal pH gradients for site-specific release.	Enables targeted colonic release Minimizes drug degradation in upper gastrointestinal tract Multi-threshold systems can adapt to individual pH variations	Significant individual pH differences affect stability Risk of premature release under acidic conditions
Charged-dependent	Exploits negatively charged mucins; surface charge modulates mucoadhesion and epithelial uptake.	Negative charge reduces nonspecific adhesion Phosphate modifications allow charge reversal to enhance uptake Ligand conjugation further improves accumulation	Charge reversal requires precise control Positive charge may lead to premature release or nonspecific adsorption
ROS-responsive	Uses elevated ROS levels (10–100× normal) in inflamed tissues to trigger drug release.	Enables precise inflammation-targeted delivery Minimizes drug exposure in healthy tissues	ROS fluctuations may affect release kinetics Prolonged ROS exposure may compromise carrier stability
Enzyme-dependent	Utilizes elevated colonic enzyme levels to degrade carriers and release drugs.	Selective response to local inflammatory enzymes Minimizes off-target delivery	Large interindividual enzyme variability Enzyme activity is influenced by the intestinal environment
Active-targeting	Targets overexpressed receptors on inflamed intestinal tissues via ligand-receptor binding.	Enhances uptake via receptor-mediated endocytosis Improves delivery precision	Ligands may cause immunogenicity Receptor expression varies among individuals Targeting depends on ligand density
Gut microbiota-targeted	Modulates gut dysbiosis via mutual interactions between nanoparticles and microbiota.	Supports microbiota rebalance for synergistic effects Reduces direct epithelial interference	Large variability in microbiota among individuals Potential disturbance of commensal flora
Barrier-targeted	Aims to restore physical, chemical, or immune barriers to disrupt inflammatory feedback.	Enhances epithelial penetration Increases drug accumulation at lesion sites Reduces systemic side effects	Penetration efficiency is low Targeting may be unstable Risk of over-repair exists

## 4. Advances in oral treatment of IBD using PNP-DDSs-loaded drugs

Recent preclinical studies have demonstrated that colonic targeting strategies utilizing PNP-DDSs enable efficient oral delivery of various anti-inflammatory agents, such as CUR, 5-ASA, and RES. These PNP-DDSs have exhibited significant therapeutic efficacy in animal models of IBD (Table 3). In this section, we review representative PNP-DDSs, discuss their mechanisms of action, and provide a comparative analysis between PNP-DDSs and existing treatment regimens.

**Table 3.** Recent advances in PNP-DDSs for oral IBD therapy.

Drugs	Carrier types	Size (S, nm); Zeta (Z, mV), PDI	Animal models	Induction methods	Design strategies	Therapeutic outcomes	Ref
CUR	HSA	S: 220.4 ± 4.3; Z: −28.80 ± 0.40; PDI: 0.102 ± 0.018	Acute UC mice	3% DSS	pH-, enzyme-dependent	Inhibits TLR4/NF-κB signaling, alleviates colitis symptoms	Luo et al., (2020)
CUR	*B. mori* SF	S: 175.4; Z: −35.5; PDI: 0.100	Acute UC mice	3.5% DSS	pH-dependent, ROS-, glutathione-responsive, and active-targeting	Maintains gut microbiota balance, alleviates colitis	Gou et al., (2019)
CUR	*B. mori* SF	S: 200; Z: −10; PDI: < 0.250	Acute/chronic UC mice	DSS	pH-dependent	Anti-inflammatory activity, significant mucosal repair	Xu et al., (2025)
CUR	LF	S: 335.60; Z: −10.44 ± 0.19; PDI: < 0.300	Acute UC mice	3% DSS	Enzyme-dependent and active-targeting	Restores mucosal barrier, maintains flora homeostasis, alleviates colitis	Ye et al., (2023)
CUR	Zein	S: 148.64 ± 3.21; Z: −15.3 ± 1.2; PDI: < 0.4	Acute UC mice	3% DSS	Active-targeting	Inhibits TLR4/NF-κB pathway, alleviates colitis symptoms	Zhang et al., (2022)
CUR	Zein	/	Acute UC mice	2.5% DSS	–	Maintains gut flora balance, alleviates colitis	Xie et al., (2024)
CUR	Zein	S: 292 ± 7.69; Z: −26.5 ± 1.47; PDI: 0.130 ± 0.02	Acute UC mice	3% DSS	–	Modulates MPO, inflammatory and oxidative factors, alleviates colitis	Zhang and Li (2024)
	Zein	S: 80 – 450; Z: < −20; PDI: 0.050 − 0.100	Acute UC mice	2% DSS	Size-, pH-dependent	Free radical scavenging, alleviates colitis symptoms	Mu et al. (2025)
RES	*B. mori* SF	S: 35 – 122	Acute IBD rats	TNBS (cannulation)	pH-dependent	Immunomodulatory and anti-inflammatory effects	Lozano-Pérez et al. (2014)
RES	*B. mori* SF	S: 170; Z: −20.5; PDI: < 0.200	Acute UC mice	3.5% DSS	pH-dependent and ROS-responsive	Anti-inflammatory and antioxidant effects	Xie et al. (2022)
RES	*A. pernyi* SF	S: 78.9; Z: −22.0; PDI: < 0.200	Acute UC mice	3.5% DSS	pH-dependent, ROS-, glutathione-responsive, and active-targeting	Regulates inflammatory microenvironment, repairs barrier, rebalances microbiota	Ma et al. (2022)
RES	*β*-lactoglobulin	S: 165; Z: −34.5; PDI: 0.12	Chronic UC Winnie mice	–	pH-dependent	Attenuates spontaneous colitis	Pujara et al. (2021)
RES	Zein	S: 174.03 ± 3.14; Z: −34.2 ± 1.15; PDI: < 0.200	Acute UC mice	3% DSS	pH-, charged-dependent	Regulates factors, balances microbiota, alleviates symptoms	Yang, Lin, et al. (2024)
CUR and RES	Zein	S: 179.83 ± 2.35; Z: −30. 83 ± 0. 74	Acute UC mice	3% DSS	pH-dependent	Regulates metabolism, balances flora, alleviates symptoms	Yang, Chen, et al. (2024)
5-ASA	HSA	S: 190; Z: −11.8; PDI: 0.35	Acute UC mice	3% DSS	Enzyme-dependent	Repairs intestinal mucosa, alleviates symptoms	Iwao et al. (2018)
PA	Gelatin	S: 243.8 ± 68.7; Z: 20.5 ± 3.3; PDI: 0.20	Acute UC mice	5% DSS	pH-dependent	Regulates biomarkers, alleviates symptoms	Ahmad et al. (2021)
PA	Hemoglobin	–	Acute UC mice	3% DSS	Enzyme-dependent	Anti-inflammatory effect, alleviates symptoms	Vaezi et al. (2024)
Que	*B. mori* SF	S: 175.8 ± 0.9; Z: −24.5 ± 4.1; PDI: 0.153 ± 0.009	Acute UC mice	3% DSS	pH-dependent	Regulates pro-inflammatory cytokines, alleviates symptoms	Diez-Echave et al. (2021)
Que	Gelatin	S: 70	Acute UC mice	3% DSS	ROS-responsive	Antimicrobial and anti-inflammatory, alleviates symptoms	Bai et al. (2025)
Que	Zein	S: 128.6 ∼ 488.0; Z: −16.5 ∼ −29.3; PDI: 0.050 − 0.525	Acute UC mice	3% DSS	pH-dependent and active-targeting	Restores barrier, microbiota, alleviates symptoms	Ma et al. (2025)
Mag	Zein	S: 142.27 ± 5.11; Z: −31.96 ± 1.72	Acute UC mice	3% DSS	Active-targeting	Regulates cytokines, repairs mucosa	Wang et al. (2021)
Mag	Zein	S: 260.5 ± 23.9; Z: −30.6 ± 1.98; PDI: 0.298 ± 0.023	Acute UC mice	3% DSS	pH-dependent	Reduces pro-inflammatory factors, alleviates symptoms	Mu et al. (2024)
MTX	HSA	S: 30 ± 3.2; Z: 37	Acute UC mice	3.5% DSS	pH-, enzyme-dependent	Regulates cytokines, alleviates symptoms	Zhang et al. (2023)
Tofacitinib	HSA	S: 250.6 ± 42.1	Acute UC mice	2.5% DSS	ROS-responsive, and active-targeting	Remodulates immune microenvironment, alleviates symptoms	Li et al. (2023)
ISL	Zein	S: 137.32 ± 2.54; Z: −37.81 ± 0.66; PDI: 0.12 ± 0.01	Acute UC mice	3% DSS	pH-, charged-dependent	Repairs mucosa, inhibits pro-inflammatory factors, alleviates symptoms	Xiao et al. (2022)
GA	Zein	S: 169.62 ± 2.73; Z: −14.99 ± 0.99; PDI: 0.159 ± 0.013	Acute UC mice	2.5% DSS	pH-, enzyme-, charged-dependent and active-targeting	Decreases MPO and TNF-α, alleviates symptoms	Wu et al. (2022)
PA	*B. mori* SF	S: 319.0 ± 23.6; Z: −30.6 ± 3.5; PDI: 0.096	Acute UC mice	3% DSS	pH-dependent and active-targeting	Modulates amino acids, regulates innate immunity, alleviates symptoms	Du et al. (2022)
EGCG	*B. mori* SF	S: 191.7 ± 6.7; Z: −24.1 ± 0.7; PDI: 0.122	Acute UC mice	3.5% DSS	pH-dependent and glutathione-responsive	Increases beneficial bacteria, alleviates symptoms	Liu et al. (2022)
Inulin and Trans-ferulic acid	*B. mori* SS	S: 81.57 ± 0.51; Z: −13.7 ± 1.75	Acute UC/Acute CD mice	2.5% DSS/2.5% TNBS	/	ROS scavenging, anti-inflammatory, promotes probiotics, alleviates IBD	Zhang et al. (2025)
PAC	*B. mori* SS	S: 136	Acute UC mice	2.5% DSS	/	Inhibits colon shortening, antioxidant/anti-inflammatory, alleviates symptoms	Wang, Li, et al. (2022)
RH	LF	S: 284.37 ± 2.70; Z: −24.9 ± 0.95; PDI: 0.163 ± 0.062	Acute UC mice	DSS	Active-targeting	Inhibits TLR4/MyD88/NF-κB, promotes healing	Luo et al. (2021)
Ferulic acid	Zein	S: > 117.6; Z: > 30; PDI: 0.5–0.6	Acute UC mice	3% DSS	/	Modulates cytokines, oxidative stress, microbiota, alleviates symptoms	Guo et al. (2025)

### 4.1. CUR-loaded PNP-DDSs

**Figure 3.**
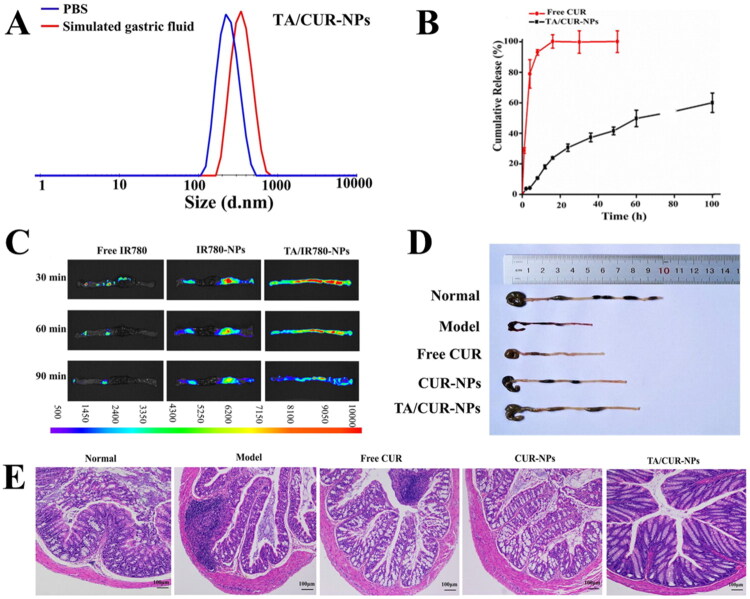
Oral administration of CUR-loaded HSA NPs for the treatment of UC mouse models. **(A)** The stability performance of TA/CUR-NPs in stimulated gastric fluids. **(B)** The release characteristics of TA/CUR-NPs under neutral conditions. **(C)** Fluorescence images demonstrating the intestinal adhesiveness of TA. **(D)** Colon length in the treatment of UC with different formulations. **(E)** H&E analysis of the Colon. *Source*: Reprinted from Luo et al. (2020) with permission. Copyright (2020), Elsevier.

CUR, a polyphenolic compound derived from turmeric, is well known for its anti-inflammatory and antioxidant properties. Its pharmacological effects are primarily mediated through the inhibition of pro-inflammatory cytokines, reduction in metalloproteinase activity, and scavenging of free radicals (Jagtap et al. 2004; Baliga et al. 2012; Mouzaoui et al. 2012; Liu et al. 2013; He et al. 2014; Jurjus et al. 2016; Lane et al. 2017). However, clinical application of CUR has been hindered by its instability and rapid clearance *in vivo*. To address these limitations, Luo et al. (2020) developed CUR-loaded HSA NPs using a combination of tannic acid encapsulation and genipin cross-linking. This formulation not only enhanced the drug encapsulation efficiency and protected CUR against degradation in simulated gastric conditions but also improved its adhesion and retention in the colonic region, thereby ameliorating symptoms in a dextran sulfate sodium (DSS)-induced UC mouse model (Figure 3).

In another approach aimed at facilitating macrophage-mediated internalization and on-demand drug release, Gou et al. (2019) engineered CUR-loaded BmSFNPs. The system exploited a multi-stimuli responsive mechanism, sensitive to pH, glutathione and ROS, with additional chondroitin sulfate modification to target the CD44 receptor on macrophages. *In vivo*, this formulation demonstrated significant accumulation in the inflamed colonic tissue, improved pharmacokinetics and bioavailability, and showed promise as a targeted therapy for UC. To overcome the challenges of poor mucus penetration and insufficient drug accumulation in colitic tissues commonly associated with oral UC therapies, Xu et al. (2025) developed CUR-loaded SFNPs modified with zwitterionic Pluronic polymers. These NPs exhibited pH-sensitive drug release behavior, significantly enhanced mucus permeability, and improved macrophage uptake efficiency. Consequently, they demonstrated potent anti-inflammatory and antioxidant effects. In a UC mouse model, the zwitterionic polymer-modified SFNPs preferentially accumulated at inflamed sites and surpassed dexamethasone in promoting mucosal healing. These findings suggest that zwitterionic polymer-modified NPs represent a highly promising platform for effective oral delivery of therapeutics in UC treatment.

A dual-targeting nanosystem developed by Ye et al. (2023) combined LF and FA with a laminarin layer to enhance targeting of both colonic epithelial cells and macrophages. This strategy improved CUR loading efficiency and promoted cellular uptake, which contributed to the alleviation of inflammation, accelerated mucosal repair, and rebalanced the intestinal microbiota.

To address the issue of NP degradation in the gastrointestinal tract, Zhang et al. (2022) designed a novel delivery system in which CUR-loaded HA/zein composite NPs were encapsulated into alginate/chitosan hydrogel microparticles via electrospraying. This system demonstrated specific targeting of macrophages, strong adhesion and retention in colonic tissues, and significantly mitigated colitis symptoms in a UC mouse model by inhibiting the TLR4/NF-κB signaling pathway. These findings highlight a promising strategy for CUR-based therapy in UC.

Recent studies have also investigated the use of tamarind seed polysaccharides as novel carriers. Xie et al. (2024) formulated CUR-loaded nanocomplexes composed of zein and tamarind seed polysaccharides via an anti-solvent method. This system significantly improved both the encapsulation efficiency and drug-loading capacity, while also restoring the intestinal microbiota and increasing the abundance of *Akkermansia* in a UC mouse model, thereby enhancing the therapeutic effect.

Additional studies have reported the formulation of zein-based biopolymer nanocomposites. For example, Zhang and Li (2024) designed a system comprising a CUR-loaded zein core with an outer coating of sodium caseinate and hyaluronic acid. This composite significantly modulated the expression of MPO, inflammatory cytokines (e.g. Tumor Necrosis Factor-α [TNF-α], Interleukin-6 [IL-6], and Interleukin-1β) and oxidative stress markers (e.g. Superoxide Dismutase, Malondialdehyde and Glutathione peroxidase), resulting in marked attenuation of UC pathology in animal models.

Furthermore, taking advantage of shellac, a biopolymer that is soluble in alkaline but not in acidic environments, Mu et al. (2025) prepared CUR-loaded composite NPs of zein and shellac using microfluidic technology. This approach improved both the biocompatibility and pH-adaptive properties of the system through precise control of particle size and dispersion. The resultant formulation exhibited pronounced antioxidant and anti-inflammatory effects in a DSS-induced UC mouse model, offering a novel perspective for IBD treatment particularly relevant to food industry applications.

Inspired by the traditional Chinese “Simmering Soup” diet, Yang, Chen, et al. (2024) developed an innovative oral nano-delivery system that employed anti-solvent precipitation combined with water evaporation cross-linking to integrate *Mesona chinensis* polysaccharides (MCP) with zein. This co-delivery system was used to encapsulate both CUR and RES. It demonstrated high encapsulation efficiency and gastrointestinal stability, effectively reduced premature drug release before reaching the colon, maintained the balance of intestinal flora, promoted short-chain fatty acid production, and alleviated UC symptoms by modulating arachidonic acid, linoleic acid, and tryptophan metabolism.

### 4.2. RES-loaded PNP-DDSs

RES is a polyphenolic compound with well-documented anti-inflammatory and antioxidant effects. Its pharmacological actions are primarily mediated by inhibiting NF-κB activation, modulating immune cell responses, and maintaining Treg/TH17 balance, in addition to scavenging ROS (Singh et al. 2012; Ren et al. 2013; Yao et al. 2015; Shi et al. 2017). Lozano-Pérez et al. (2014) developed RES-loaded BmSFNPs that exploited the intrinsic anti-inflammatory properties of *B. mori* SF. Their formulation effectively reduced pro-inflammatory cytokines and promoted colonic mucosal repair in a murine model, achieving efficacy comparable to dexamethasone.

To overcome RES’s inherent poor water solubility and low bioavailability, Ma et al. (2022) engineered RES-loaded NPs using modified *A. pernyi* SF. This system not only targeted colonic epithelial cells and macrophages but also exhibited lysosomal escape capabilities and responsiveness to pH, ROS, and glutathione. Therapeutic evaluations demonstrated restoration of the colonic epithelial barrier, promotion of macrophage polarization toward the anti-inflammatory M2 phenotype, and marked reductions in both inflammatory responses and intracellular ROS, thereby effectively ameliorating UC symptoms.

Xie et al. (2022) addressed NP retention within the mucus layer by developing Pluronic F127-functionalized, RES-loaded BmSFNPs. The modification enhanced mucus permeability and significantly inhibited the secretion of TNF-α and ROS by RAW 264.7 macrophages under lipopolysaccharide stimulation. Furthermore, leveraging the hydrophobic pocket of *β*-lactoglobulin, Pujara et al. (2021) formulated *β*-lactoglobulin-encapsulated RES nanocarriers that substantially improved both solubility and release efficiency *in vitro* and *in vivo*, yielding significant alleviation of IBD symptoms and a reduction in the disease activity index. Additionally, Yang et al. utilized MCP-modified ZNPs to encapsulate RES (Yang, Lin, et al. 2024). This strategy enhanced RES’s water solubility and colonic aggregation, remodeled the mucus layer, modulated the balance of pro- and anti-inflammatory mediators, and normalized the intestinal microbiota, leading to marked symptomatic relief in a murine UC model.

### 4.3. 5-ASA-loaded PNP-DDSs

5-ASA, also known as Mesalamine, exerts its anti-inflammatory activity primarily by inhibiting inflammatory mediators, blocking key signal transduction pathways, reducing cytokine production, and scavenging free radicals (Sharon et al. 1978; Stenson and Lobos 1982; Ahnfelt-Rønne et al. 1990; Shanahan et al. 1990; Stenson 1990; Mahida et al. 1991; Cominelli et al. 1992; Rachmilewitz et al. 1992; Rogler et al. 1998; Qureshi and Cohen 2005). Recognizing that IBD is often associated with elevated levels of MPO at inflammatory sites, Iwao et al. (2018) developed a 5-ASA-loaded HSA NPs system. This formulation exploited the specific interaction between MPO and HSA to effectively penetrate the intestinal mucus layer, thereby localizing drug release to inflamed regions. In murine UC models, this approach significantly reduced both the disease activity index and intestinal tissue damage.

Given the resistance of Eudragit-S100 to gastric acid, Ahmad et al. (2021) designed an enteric-coated gelatin NP system loaded with 5-ASA. This enteric coating protected 5-ASA from degradation in the stomach, ensured precise delivery to the colon, and demonstrated pronounced anti-inflammatory effects along with good biocompatibility in a dextran sulfate sodium-induced IBD mouse model. Moreover, building on previous findings that hemoglobin can serve as an effective drug carrier through its interaction with MPO, Vaezi et al. (2024) developed 5-ASA-loaded hemoglobin NPs. This formulation, which benefited from strong adhesion properties, achieved targeted delivery and prolonged drug retention in the colon, resulting in effective alleviation of colonic inflammation in a murine UC model.

### 4.4. Quercetin-loaded PNP-DDSs

Quercetin (Que), a flavonoid abundantly present in fruits and vegetables, modulates the development of IBD through pathways such as EPK1/2–FKBP and RXR–STAT3, thereby exerting both anti-inflammatory and antioxidant effects (Dong et al. 2020). However, Que is prone to premature absorption and degradation in the stomach and small intestine, which severely limits its bioavailability. To address this limitation, Patricia et al. employed BmSFNPs to encapsulate Que, demonstrating that this formulation effectively restored the mucosal barrier and reduced pro-inflammatory cytokine levels in a DSS-induced murine model (Diez-Echave et al. 2021). This strategy overcame early degradation and preserved the flavonoid’s intrinsic anti-inflammatory activity, suggesting considerable potential for clinical application of Que.

Subsequently, Bai et al. (2025) developed GNPs loaded with Que and coated with dopamine, which possesses inherent gastroprotective properties. This novel system not only enhanced Que’s solubility and bioavailability but also achieved controlled drug release while maintaining robust anti-inflammatory effects, thereby making Que-based therapy more promising. In another approach, Ma et al. (2025) formulated co-loaded NPs using egg white-derived peptides and Que on a zein/chondroitin sulfate template. The incorporation of egg white-derived peptides enhanced Que’s solubility and stability, while chitosan provided active-targeting capabilities, leading to effective repair of the intestinal barrier and restoration of intestinal microbiota balance.

### 4.5. PNP-DDSs loaded with other drugs

Magnolol (Mag), a major bioactive compound extracted from *Magnolia officinalis*, exhibits anti-inflammatory properties and promotes mucosal healing by modulating the MAPK, NF-κB, and PPAR-γ signaling pathways (Shen et al. 2018). However, like many natural phytochemicals, Mag suffers from poor water solubility, low chemical stability, rapid metabolism, and fast systemic elimination, which limit its clinical application (Chen et al. 2019). To overcome these limitations, Wang et al. (2021) developed Mag-loaded ZNPs coated with chondroitin sulfate and embedded them into hydrogel microspheres via electrospraying. This system effectively alleviated colonic inflammation by rebalancing pro- and anti-inflammatory cytokines and repairing the colonic mucosal barrier through upregulation of tight junction proteins, such as ZO-1 and occludin. In another approach aimed at prolonging Mag release and minimizing systemic exposure before reaching the colon, Mu et al. (2024) encapsulated Mag-loaded ZNPs within a three-layer polyelectrolyte shell composed of polydopamine–chitosan and cellulose acetate phthalate. This formulation exhibited pH-responsive and sustained-release characteristics. In a UC mouse model, it significantly suppressed pro-inflammatory cytokine production, demonstrating robust anti-inflammatory efficacy.

Methotrexate (MTX), an antimetabolic agent with anti-inflammatory properties, functions primarily by inhibiting dihydrofolate reductase and interacting with cell-surface adenosine receptors (Cronstein et al. 1983, 1985, 1992, 1993; Schröder and Stein 2003). Given the high affinity between HSA and MTX, Zhang et al. (2023) designed an innovative oral drug delivery platform wherein MTX was encapsulated in HSA NPs and subsequently enclosed within multilayer microcapsules composed of calcium alginate and chitosan. This multi-protection system effectively shielded MTX from degradation in gastric acid and protease-rich environments, ensured precise colonic delivery, significantly reduced systemic toxicity, and demonstrated excellent safety and efficacy in UC models.

Tofacitinib, a Janus kinase (JAK) inhibitor that impedes the JAK1, JAK2, and JAK3 signaling pathways to reduce inflammatory mediator release, is limited clinically by its high toxicity and adverse side effects (Jiang et al. 2008; Weisshof et al. 2018; Mishra et al. 2022). To mitigate these issues, Li et al. (2023) developed a ROS-responsive HSA NP system modified with an anti-MAdCAM-1 antibody. This formulation achieved targeted delivery to the colon, prolonged drug retention, promoted macrophage polarization toward the anti-inflammatory M2 phenotype, and inhibited pro-inflammatory cytokine production, effectively alleviating the inflammatory response in UC.

**Figure 4.**
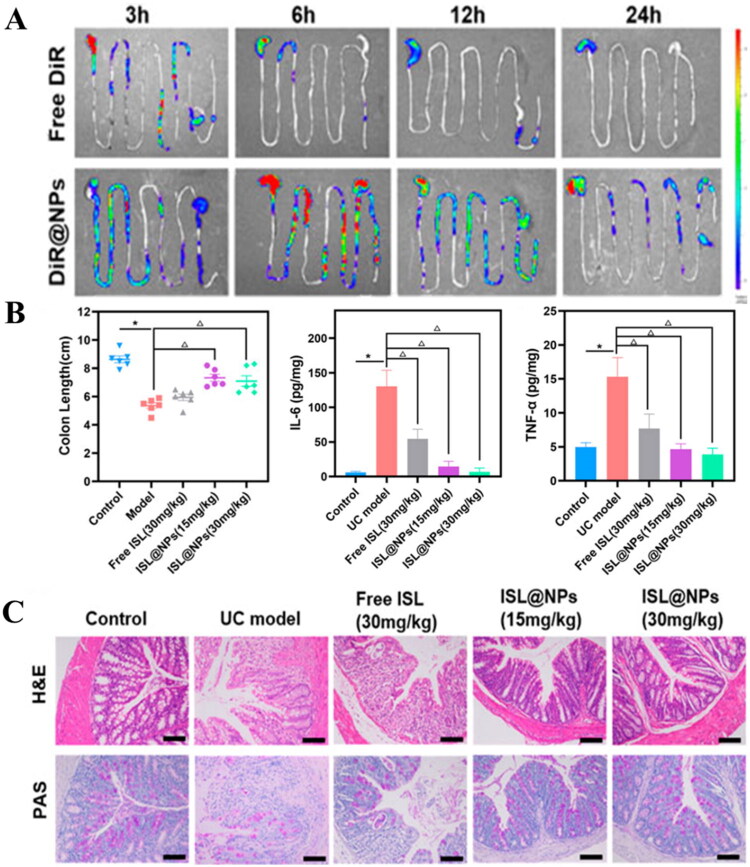
Oral administration of ISL-loaded Zein NPs for the treatment of UC mouse models. **(A)** Fluorescence images showing the *in vivo* distribution and retention time of ISL@NPs. **(B)** Colon length, IL-6 levels, and TNF-α levels under treatment with different formulations (or concentrations). **(C)** H&E analysis of the Colon under treatment with different formulations (or concentrations). Reproduced under terms of the CC-by license. *Source*: Xia et al. published by Frontiers. Copyright (2022).

Isoliquiritigenin (ISL), a natural anti-inflammatory compound, suffers from poor solubility, instability, and rapid clearance. Xiao et al. (2022) addressed these challenges by preparing ISL-encapsulated NPs composed of zein and caseinate via an anti-solvent method. This approach enhanced the stability and targeted delivery of ISL, prolonged its retention in the colon, minimized drug loss in the upper gastrointestinal tract, promoted the repair of damaged colonic mucosa and suppressed pro-inflammatory factor expression, thereby improving therapeutic outcomes in UC models (Figure 4).

Glycyrrhizic acid (GA), an active ingredient extracted from *Glycyrrhiza glabra*, exhibits potent anti-inflammatory effects but is hampered by low bioavailability and instability. Wu et al. (2022) developed a nanocarrier system comprising a FA-modified zein core enveloped by a pectin shell. This system maintained high stability in both gastric and small intestinal environments and released GA upon pectin degradation in the colon. The targeted delivery reduced the expression of inflammatory mediators such as MPO and TNF-α in immune cells, thereby significantly ameliorating IBD symptoms and demonstrating high therapeutic potential.

Patchouli alcohol (PA), a naturally derived sesquiterpenoid from patchouli, effectively mitigates inflammation and repairs intestinal barrier damage by reducing pro-inflammatory cytokine expression and upregulating tight junction proteins and mucins (Wu et al. 2020). Du et al. (2022) developed BmSFNPs loaded with PA and functionalized their surface with a cyclic RGD peptide, which targets integrin receptors on intestinal epithelial cells. Both *in vitro* and *in vivo* evaluations revealed that this system significantly enhanced targeted drug delivery, inhibited inflammatory responses, modulated amino acid metabolism, and facilitated the repair of intestinal damage, offering a novel strategy for targeted therapy in UC.

Epigallocatechin-3-gallate (EGCG), the predominant catechin in green tea, possesses substantial antioxidant properties and modulates inflammation via multiple signaling pathways (Singh et al. 2011). Liu et al. (2022) successfully formulated EGCG-loaded BmSFNPs and enhanced macrophage uptake through surface modification with Cathelicidin-BF, which imparts a positive surface charge. The resultant formulation effectively decreased pro-inflammatory cytokine levels, increased anti-inflammatory factor secretion, promoted M2 macrophage polarization, and scavenged lipopolysaccharides. Furthermore, when administered orally in a hydrogel matrix, these NPs significantly enriched gut microbial diversity and enhanced therapeutic efficacy in UC models.

Orally administered probiotics can exert therapeutic effects in IBD by regulating gut microbiota and intestinal immune function. However, probiotics often suffer reduced efficacy due to susceptibility to destructive gastrointestinal factors such as gastric acid, digestive enzymes, and excessive ROS produced in inflamed tissues (Liu, Li, et al. 2021; Wu and Liu 2022). To enhance the therapeutic efficacy of oral probiotics, Zhang et al. (2025) developed BmSSNPs loaded with inulin (to promote probiotic proliferation) and trans-ferulic acid (possessing antioxidant activity. They then constructed a protective polymeric barrier composed of the cationic polymer-poly-lysine and anionic polymer-poly-glutamic acid on the surface of *Bifidobacterium longum* probiotics via electrostatic adsorption. This protective barrier effectively shielded probiotics from destructive gastrointestinal factors. The resulting oral delivery system for *B. longum* demonstrated excellent gastrointestinal resistance, along with notable antioxidant, anti-inflammatory, and intestinal barrier-repairing effects in mouse models of UC and CD. Furthermore, the synergistic interaction between the polyelectrolytes and inulin significantly enhanced mucosal adhesion and probiotic proliferation, thereby substantially improving the overall therapeutic outcomes of orally administered probiotics.

Proanthocyanidins (PACs), oligomers composed of flavan-3-ol monomers widely distributed in fruits and vegetables (Jiao et al. 2017), have demonstrated anti-inflammatory, antioxidant, and anti-apoptotic activities, as well as inherent colon-targeting properties beneficial for alleviating UC (Cheah et al. 2013). However, their poor aqueous solubility and low stability have significantly limited their clinical application. To address these issues, Wang, Li, et al. (2022) developed a BmSSNP delivery system to encapsulate PACs . In a DSS-induced UC mouse model, this NP delivery system exhibited excellent biocompatibility and significantly improved UC symptoms by effectively preventing colon shortening, mitigating oxidative stress and inflammation, and promoting repair of damaged intestinal tissue.

Rhein (RH), a bioactive compound extracted from *Rheum palmatum* L., exerts potent anti-inflammatory effects by modulating multiple signaling pathways, including NF-κB, JAK/STAT, and MAPK (Henamayee et al. 2020). It also provides protective benefits for the intestinal mucosal barrier (Sun et al. 2016). However, its clinical translation has been hindered by poor water solubility, low bioavailability, and inadequate colon-targeting capacity (Wei et al. 2017). To address these limitations and harness the full therapeutic potential of RH, Luo et al. (2021) developed RH-loaded LFNPs modified with calcium pectinate and HA. This dual-modified nanosystem effectively protected RH from degradation in the harsh gastrointestinal environment, facilitated targeted delivery to colonic lesion sites, and significantly attenuated inflammation by inhibiting the TLR4/MyD88/NF-κB signaling pathway. The resulting colonic healing effects highlight the promise of this strategy in developing food-derived nanomedicines for UC treatment.

Ferulic acid, one of the most abundant phenolic acids found in cereals, has demonstrated therapeutic potential for UC by modulating gut microbiota, enhancing the expression of tight junction proteins in intestinal epithelial cells, and increasing the population of regulatory T cells (Katayama et al. 2017; Tian et al. 2022; Wang, Tang, et al. 2022; Kim et al. 2024). However, its clinical efficacy is limited due to its susceptibility to degradation by environmental factors and gastrointestinal enzymes. To overcome these challenges and improve its biological stability, Guo et al. (2025) designed a novel zein/ferulic acid–pectin/chitosan nanocomplex. This multifunctional delivery system significantly alleviated UC symptoms in murine models by modulating inflammatory cytokine production, reducing oxidative stress, and restoring gut microbiota balance. The findings highlight the promise of this nanocomplex as a targeted oral delivery platform for UC therapy.

### 4.6. Comparison of PNP-DDSs and existing treatment regimens

Current IBD therapies primarily aim to alleviate symptoms but often face limitations such as poor targeting efficiency, suboptimal drug concentrations at intestinal sites, limited efficacy in treating chronic lesions, and progressive loss of therapeutic response. In contrast, PNP-DDSs leverage the targeting capabilities of protein-based carriers to achieve precise drug delivery to inflamed regions, significantly enhancing local drug concentrations and anti-inflammatory effects—particularly in complex or refractory lesions. In terms of safety, conventional treatments are frequently associated with adverse effects: corticosteroids can lead to osteoporosis, immunosuppressants increase susceptibility to infections, and biologic agents may provoke allergic reactions. PNP-DDSs mitigate these issues by reducing systemic exposure and utilizing biocompatible, low-toxicity carriers. Regarding patient compliance, many current therapies require inconvenient routes of administration (e.g. injections, rectal suppositories) or frequent dosing, which may reduce adherence. PNP-DDSs enable oral administration with prolonged release profiles, allowing for reduced dosing frequency and improved patient compliance. Overall, PNP-DDSs demonstrate significant advantages over existing therapeutic approaches, as summarized in Table 4.

**Table 4.** Comparative analysis of PNP-DDSs and current therapeutic strategies for IBD.

Comparison dimension	Conventional IBD therapies	PNP-DDSs
Efficacy	Poor targeting capability. Low drug concentration at the site of inflammation. Limited efficacy in treating chronic or refractory lesions. Diminished efficacy over time.	High colonic targeting precision. Enhanced local drug accumulation. Improved therapeutic effect in complex or persistent lesions. Sustained and long-lasting efficacy.
Safety	Higher incidence of adverse effects. Glucocorticoids may induce osteoporosis. Immunosuppressants increase susceptibility to infections. Biologics may cause systemic immune reactions.	Reduced systemic exposure, minimizing toxicity and side effects. Biodegradable and biocompatible materials. Low immunogenicity. Lower risk of systemic damage from long-term use.
Patient compliance	Low adherence due to intravenous or rectal routes of administration. Oral formulations often require frequent dosing due to instability in the intestinal environment.	Resistant to enzymatic degradation and harsh pH conditions in the Gastrointestinal Tract tract. High drug stability. Reduced dosing frequency. Improved patient adherence and acceptance of long-term therapy.

## 5. Challenges and prospects

PNP-DDSs have shown considerable promise for the oral treatment of IBD. However, several key challenges must be addressed to facilitate their clinical translation:

### 5.1. Clinical heterogeneity

Patients with IBD exhibit substantial heterogeneity driven by factors such as genetic polymorphisms (e.g. NOD2 mutations), dynamic imbalances in the gut microbiota and spatial as well as temporal variations in inflammatory pathology. These factors result in significant fluctuations in the colonic microenvironment. For example, in UC patients, focal pH values may vary from 5.5 to 7.5, whereas in CD patients, pH can range more broadly from 3.0 to 7.5 (Zeeshan et al. 2019). Currently, most pH-dependent PNP-DDSs are engineered around a single threshold (typically pH 6.0), which yields a static response mechanism. This approach is inadequate to accommodate individual differences and dynamic changes during disease progression. Future research should focus on: (1) developing drug delivery systems with multi-threshold dynamic response capabilities; (2) designing platforms tailored specifically for UC and CD; (3) utilizing artificial intelligence to continuously adjust the response parameters for precise and adaptive drug release.

### 5.2. Limitations of single-stimulus systems

Existing PNP-DDSs often rely on single-stimulus response mechanisms (e.g. pH-dependent systems) that are susceptible to either premature or delayed drug release due to dramatic pH fluctuations in the intestinal tract. Emerging strategies, such as the development of multimodal intelligent response systems, have the potential to mitigate these issues by synergistically regulating multiple trigger thresholds. Recent studies have shown that CUR-loaded BmSFNPs incorporating a pH/ROS dual response mechanism can enhance colonic targeting (Gou et al. 2019). Nonetheless, further optimization is required to improve their scalability and stability. Similarly, charged-dependent nanosystems are largely designed with irreversible charge responses. A reversible charge-switching strategy responsive to factors such as pH and enzymatic activity could facilitate both effective penetration through the mucosal barrier and subsequent cellular uptake. In addition, at inflammation sites with elevated ROS or protease levels, self-degradable delivery shells capable of adaptive disintegration into smaller fragments may enhance NPs permeability and prolong drug retention at the target site. Integrating techniques such as charge modulation or ligand modification with advanced microfluidic chip technology and process analytical technology could also improve the consistency and scalability of nanocarrier production.

### 5.3. Challenges in clinical translation

PNP-DDSs have achieved clinical breakthroughs in various diseases beyond IBD. For instance, albumin-bound paclitaxel (Abraxane^®^) has been approved in multiple countries, including the USA, European Union, and Japan, for metastatic breast cancer, locally advanced or metastatic non-small cell lung cancer, and pancreatic adenocarcinoma; Japan has further approved its use for advanced gastric cancer (Desal 2016). However, there have been no clinical reports regarding PNP-DDSs in IBD treatment to date. Translating PNP-DDSs from mouse models of IBD to human clinical practice faces substantial complexities, outlined as follows:

1. Pathophysiological differences between animal models and human IBD
  Mouse models typically exhibit chemically induced acute inflammation (e.g. DSS-induced barrier disruption), whereas human IBD is a chronic, multifactorial disease driven by genetic susceptibility, microbiota dysbiosis, and environmental influences, coupled with significant individual variability. Furthermore, the anatomical and physiological differences, such as intestinal length, transit time, and peristaltic frequency, between mice and humans complicate direct extrapolation of NP retention time and interactions with gut microbiota from preclinical models to patients.
2. Cross-species differences in biocompatibility and safety
  Immunogenicity poses a critical challenge due to the differing immunological tolerances between mice and humans toward heterologous proteins. For example, SF, which rarely triggers antibody responses in mice, could be recognized as a foreign antigen by humans, potentially causing IgE-mediated allergic reactions or complement activation, risks especially heightened with prolonged administration. Additionally, long-term toxicity and metabolite accumulation are concerns: mouse experiments typically last 4–8 weeks, while human IBD management requires chronic treatment spanning months or years. Rapidly cleared protein degradation products (amino acids, short peptides) in mice may accumulate over time in humans, potentially impairing intestinal nutrient absorption and imposing additional burden on hepatic and renal functions.
3. Bottlenecks in clinical translation of targeted delivery efficiency
  The species-specific nature of targeting ligands presents a significant barrier. NPs used in preclinical studies often target markers abundantly expressed at inflammatory sites in mouse intestines; however, these markers may exhibit different expression patterns or lower expression levels in humans, thereby reducing targeting efficiency. Additionally, differences in intestinal blood flow and barrier properties, such as vascular density and permeability, between humans and mice result in reduced NP accumulation at inflamed sites via the enhanced permeability and retention effect. Other practical barriers include challenges in achieving large-scale production, maintaining quality control standards, and navigating regulatory pathways and clinical trial design.

Proposed strategies to overcome these barriers include the following: (1) Developing animal models more representative of human IBD, such as humanized gut microbiota or genetically engineered mice. (2) Optimizing NP designs, including employing composite polymer coatings for enhanced stability and incorporating dual-targeting ligands to improve specificity. (3) Establishing clinical-grade manufacturing processes, such as microfluidic-based techniques for achieving consistent NP size distribution and quality. (4) Conducting small-dose, multi-center early-phase clinical trials prioritizing safety assessments over efficacy outcomes.

Only through systematically addressing these translational challenges can PNP-DDSs progress effectively from bench to bedside, ultimately offering novel, targeted therapeutic options for patients suffering from IBD.

### 5.4. Model limitations

The pathogenesis of IBD is multifactorial, involving intestinal epithelial injury, immune dysregulation, and gut microbial dysbiosis. An ideal preclinical model should accurately replicate these features to facilitate mechanistic research and therapeutic development. However, commonly used animal models exhibit significant limitations in recapitulating the complexity of human IBD. For example, the widely utilized DSS-induced model triggers intestinal inflammation primarily by chemically disrupting epithelial barrier integrity through direct toxicity to enterocytes. While valuable for studying epithelial damage, this approach lacks key features of immune dysregulation—a defining characteristic of human IBD. In human patients, the activation of both innate and adaptive immunity drives disease progression, whereas DSS-induced inflammation arises secondarily to epithelial damage, failing to reproduce the complex immune regulatory dysfunction. Additionally, DSS-induced inflammation tends to be acute and self-limiting, inadequately representing the chronic, relapsing nature of IBD observed clinically.

To more effectively model UC and CD, we recommend employing chronic or immune-mediated models that better mimic immune dysregulation and disease chronicity. For instance, IL-10 knockout mice develop spontaneous, chronic intestinal inflammation due to impaired immune regulation, closely replicating the immunopathological aspects of human IBD. Similarly, T-cell transfer models—involving adoptive transfer of CD4^+^CD45RB^high^ T cells into immunodeficient hosts (such as *Rag1*^-/-^ mice)—produce robust, antigen-specific immune responses, emphasizing the central role of adaptive immunity in IBD pathogenesis.

In summary, while DSS-induced models are useful for studying mechanisms of epithelial injury, their limited ability to reflect immune dysregulation restricts translational relevance. Integrating immune-driven models, including IL-10 knockout and T-cell transfer approaches, would enhance preclinical applicability, providing more comprehensive platforms to elucidate IBD pathogenesis and assess novel therapeutic interventions.

### 5.5. Manufacturing and regulatory challenges

PNP-DDSs face substantial manufacturing and regulatory barriers in their clinical translation. From a production standpoint, the lack of standardized NP preparation protocols presents a major challenge. Techniques such as emulsion–solvent evaporation or self-assembly are widely used, but procedural inconsistencies across laboratories and manufacturing facilities, due to the absence of unified industrial standards, lead to variability in key physicochemical properties (e.g. particle size, surface charge, drug loading). This, in turn, compromises the reproducibility of biological performance and hinders data comparability and integration.

Another significant issue is batch-to-batch variability. Small fluctuations in raw material purity, reaction temperature, pH, or agitation speed can cause notable differences in NP structure and function. For instance, variations in the folding or surface modification of protein carriers can affect targeting efficiency and drug release profiles, resulting in inconsistent therapeutic outcomes and heightened clinical risk. In essence, the transition from laboratory-scale production to good manufacturing practice-compliant large-scale manufacturing is hindered by the difficulty of replicating the precise control achievable in small-scale experiments. Additionally, maintaining protein stability, purity, and process consistency during scale-up remains a significant challenge.

Regulatory challenges further complicate clinical translation. Existing regulatory frameworks are primarily designed for conventional small-molecule drugs or biologics and are poorly suited to address the complex nature of PNP-DDSs. Their unique physicochemical characteristics, *in vivo* metabolic behavior, and uncertain long-term toxicity profiles are not adequately covered by current guidelines. Moreover, conventional toxicity assessment methods fail to capture NP-specific issues such as intracellular accumulation and immunogenicity. Critical quality attributes, such as particle size distribution and carrier degradation rate, also lack standardized definitions and benchmarks. These gaps in regulatory infrastructure contribute to prolonged approval timelines and represent a major hurdle for the clinical adoption of PNP-DDSs.

### 5.6. Safety and ethical considerations

Potential safety risks represent a significant challenge for PNP-DDSs. Animal-derived proteins, such as BSA, raise concerns about prion contamination, which can lead to irreversible neurological damage, as well as the possibility of immune rejection. Even natural proteins generally regarded as low-immunogenic, such as HSA and gelatin, may still provoke immune responses in certain individuals due to genetic or immunological variability. In addition, ethical issues related to animal welfare, alongside the high costs and strict biosafety regulations associated with large-scale animal protein production, limit the feasibility of meeting long-term global demand for IBD treatments. In contrast, plant-derived proteins like zein offer several advantages—they are free from prion contamination, highly biocompatible, and more environmentally sustainable. However, their inherent hydrophobicity can reduce drug-loading efficiency. Future directions to address these challenges may include: (1) incorporating amphiphilic block peptides to fine-tune the carrier’s amphiphilicity; (2) utilizing microfluidic technologies for precise NP fabrication; and (3) applying synthetic biology and immunogenicity modification techniques to minimize or eliminate immune responses, thereby improving clinical compatibility and efficacy.

### 5.7. Application of combination therapy

The development of PNPs capable of delivering multiple drugs simultaneously, or integrating NP delivery with biotherapies, holds significant promise for IBD treatment. PNPs, with their inherent biocompatibility and structural versatility, can precisely encapsulate agents with different mechanisms of action, enabling synergistic release at the disease site. For example, ZNPs co-loaded with CUR and RES have demonstrated enhanced therapeutic efficacy in IBD models. Moreover, the combination of NP-mediated drug delivery with biotherapeutic approaches expands the therapeutic landscape. For instance, NPs carrying anti-inflammatory agents can penetrate the intestinal inflammatory microenvironment, while co-delivered probiotics help restore microbial balance. Additionally, NPs can be engineered to deliver biologics such as anti-TNF-α monoclonal antibodies or anti-integrin agents directly to inflamed sites, establishing a “delivery–regulation–anti-inflammation” loop. This integrated approach offers a promising strategy to overcome the limitations of monotherapies and achieve more effective, targeted treatment for IBD.

In summary, while PNP-DDSs represent a transformative approach to IBD treatment, overcoming these challenges through multidisciplinary collaboration and technological innovation is essential for their successful clinical translation. Over the next 5 years, we anticipate that PNP-DDSs will expand their range of protein sources and integrate novel technologies and modifications to address existing limitations. Concurrently, new targeting strategies will be developed, based on the pathophysiological characteristics of IBD, to significantly improve therapeutic efficacy, potentially ushering in a new era of effective, oral IBD treatments.

## 6. Conclusion

PNP-DDSs have emerged as a pivotal research focus in the oral treatment of IBD due to their capacity to enhance drug stability, targeting specificity, bioavailability, and controlled release. Despite promising outcomes observed primarily in preclinical UC models, clinical data remain limited, underscoring the need for further investigation to fully validate their efficacy and safety in human patients. Future research should prioritize the clinical translation of PNP-DDSs by optimizing their dynamic response mechanisms, improving formulation stability and scalability and advancing safety assessments through multidisciplinary collaboration and cutting-edge technological innovations. Given the complex pathophysiology of IBD and ongoing advancements in nanoparticle engineering, it is anticipated that PNP-DDSs will increasingly contribute to more effective, safe, and precisely targeted therapeutic strategies for IBD management.

## Data Availability

Data sharing not applicable.
